# Spacer-based gap balancing is useful in total knee arthroplasty: a 3-year follow-up of a retrospective study

**DOI:** 10.1186/s13018-021-02788-6

**Published:** 2021-10-21

**Authors:** Yanhui Hu, Da Song, Yi Liu, Yong Zhao, Wenpu Ma, Yiqun Yang, Zhenfeng Yuan

**Affiliations:** 1 Department of Orthopaedics, Liaocheng People’s Hospital, 67 Dongchang West Road, Liaocheng, 252000 Shandong China; 2 Department of Orthopaedics, Linqing People’s Hospital, Liaocheng, Shandong China

**Keywords:** Total knee arthroplasty, Gap balancing, Measured resection technique, Spacer, Ligament balancing

## Abstract

**Background:**

Which technique, gap balancing or measured resection, can obtain better femoral component alignment and soft tissue balance in total knee arthroplasty (TKA) is still controversial. This study aimed to determine whether the gap balancing technique using a modified spacer block in TKA can result in better postoperative clinical outcomes than the measured resection technique.

**Methods:**

A total of 124 patients who underwent consecutive primary TKA between May 2016 and August 2018 were retrospectively reviewed. The gap balancing technique assisted by a modified spacer block was used in 61 patients, and the measured resection technique was used in 63 patients. The surgical, imaging and knee function outcomes of the two groups were compared.

**Results:**

The thickness of the posterior medial condyle bone resection using the modified spacer block tool in gap balancing was significantly larger than that of the MR technique (*P* = 0.001). Compared with the measured resection group, the gap balancing group had a greater external rotation resection angle of the femur (4.06 ± 1.10° vs. 3.19 ± 0.59°, *P* < 0.001°). Despite these differences, the mean ROM, KSS scores, and WOMAC scores at the 6-week, 1-year, and 2-year follow-ups were not significantly different. Postoperatively, there was no significant difference between the two groups in mechanical axis measurements (*P* = 0.275), the number of HKA outliers (*P* = 0.795) or the joint line displacement (*P* = 0.270).

**Conclusion:**

The functional outcomes of the gap balancing technique based on the modified spacer are similar to those of measured resection at 3 years. Compared with the MR technique, the GB technique resulted in a greater external rotation resection angle and thicker posterior medial condylar cuts in TKA with knee varus.

## Introduction

Total knee arthroplasty (TKA) has been established as a safe and effective surgical treatment for patients with severe knee osteoarthritis. Despite its successful clinical benefits, 19% of patients still suffer from poor joint balance [1] and instability [2] that cause postoperative pain, decreased patient satisfaction and might require revision surgery [3, 4]. The precise positioning and alignment of the prosthesis in the coronal and sagittal planes and the balance of the soft tissues are critical to the recovery of function after TKA [5, 6]. Gap balancing (GB) and measured resection (MR) are two different surgical techniques that can be used to achieve this goal [5, 7–10].

The MR technique uses anatomical markers such as the transepicondylar axis (TEA), anteroposterior (AP) axis, or the posterior condylar axis (PCA) to determine the rotation of the femoral prosthesis [5, 7, 11]. However, some researchers have shown that MR technology has great variability in setting the rotation of the femoral components due to individual differences in anatomical landmarks [12, 13]. In addition, it has been reported that MR technology has caused an increased incidence of femoral condyle lift-off, which may potentially lead to implant instability [8, 14].

Conversely, the GB technique relies on the optimal soft tissue tension to obtain equal and balanced extension and flexion gaps. Previous studies have shown that TKA with GB technology can achieve good femoral rotation alignment and flexion stability [13, 15, 16]. However, the GB technique can only be completed with the aid of specific tools during its implementation. The tensioner-based gap balancing technique is one of the most frequently used gap-balancing techniques, but it fails to reproduce the physiologic varus laxity of the knee in flexion [17], and it is also difficult to accurately control the distraction force during its application [18].

Although computer-assisted navigation (CAS) and patient-specific instrumentation (PSI) techniques can achieve better prosthesis alignment and joint line repair, they are inferior in improving the rotational alignment of the femur [19, 20]. In recent years, some researchers have suggested that the implementation of a gap balancing technique based on spacer blocks can achieve a natural knee ligament balance, and it also has the characteristics of a low technical cost, a low cost, and good reproducibility [17, 21, 22].

**Fig. 1:**
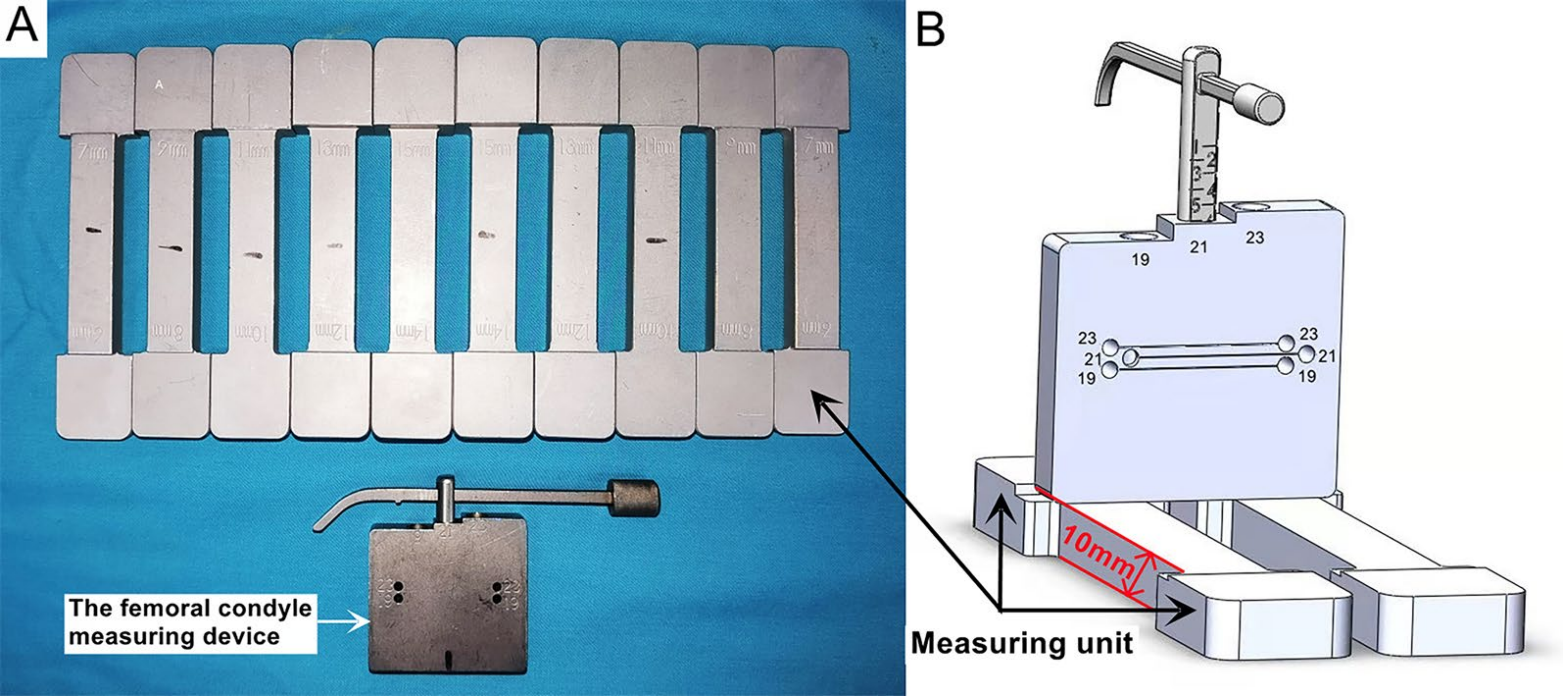
Modified spacer block tool for gap balancing (**A**) and 3Dmax drawings (**B**). The modified spacer block is a dumbbell-shaped metal module with a flat bottom, comprised of a handle with a thickness of 10 mm in the middle and measuring units at both ends with a measuring gap range of 6–15 mm. The femoral condyle measuring device was specially designed as a posterior reference aiming system with nail holes marked at 19, 21, 23 to represent the obtained flexion gap (19 mm, 21 mm, 23 mm), respectively. Its accuracy is 1 mm

The purpose of this study was to: (1) introduce a modified spacer block tool (Fig. 1) to perform flexion gap balancing and (2) to evaluate the surgical and radiographic parameters, complications, and patient outcomes of patients receiving this GB technique compared with the MR technique.

## Patients and methods

### Ethical approval

This study was approved by the institutional ethics committee, and each participant signed an informed consent form.

### Study design

Between May 2016 and August 2018, 124 consecutive patients underwent primary TKA with one of two surgical techniques: the GB technique assisted by a modified spacer block (*n* = 61) or the traditional MR technique (*n* = 63). The inclusion criteria were patients who were 50–75 years old with primary knee osteoarthritis with knee varus, who had a poor response to conservative treatment and a severely impaired ability to perform daily activities, and who had participated in a systematic follow-up for at least 3 years. All patients underwent a preoperative physical examination (to determine the collateral ligament integrity through varus and valgus stress testing with the knee at 20° of flexion) and radiographic evaluation (standing full-length anteroposterior and knee lateral X-rays). Patients who did not want to participate in the study or who had collateral ligament dysfunction and knee varus > 20° were excluded. There were no significant differences in baseline patient demographics between the two groups in terms of age, sex, side of surgery or preoperative BMI (Table 1).

**Table 1:** Patient demographics

	GB group (*n* = 61)	MR group (*n* = 63)	*P* value
Age (years)	64.89 ± 5.91	66.19 ± 4.87	0.182
Gender (female/male)	38/23	42/21	0.611
Side (left/right)	25/36	24/39	0.742
BMI (kg/m^2^)	26.38 ± 2.73	26.89 ± 2.79	0.308

*BMI* Body mass index

### Surgical procedure

All surgeries were performed by the same senior knee arthroplasty surgeon using a posterior-stabilized TKA prosthesis (XN, Chunlizhengda Medical Instruments Co. Ltd., Beijing, China). Both groups used a median anterior knee incision with a medial parapatella approach.

After the knee was exposed, the osteophytes were removed, and the same intramedullary and extramedullary guidance systems were used to perform distal femoral and proximal tibial resections. Then, the GB and MR techniques were used for the femoral rotation resection.

### GB group

**Fig. 2:**
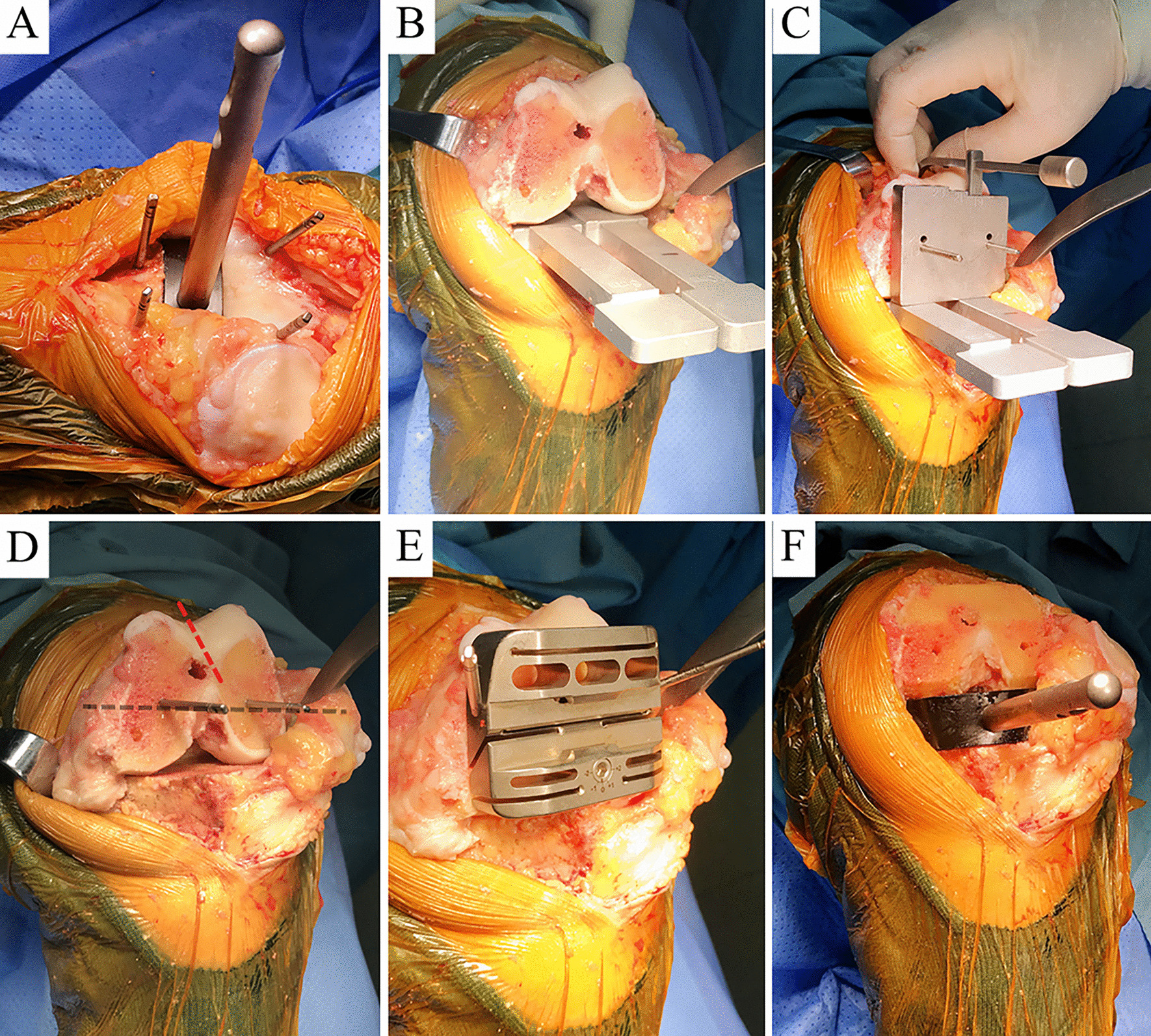
Surgical technique (**A**–**F**). **A** The extension gap was evaluated for size and balancing by a traditional spacer block. **B** The modified spacers were inserted in the joint space of the knee at 90° of flexion, and the tension of the medial and lateral ligaments were balanced. **C** The condyle measuring device determines the size of the femoral prosthesis and the AP position of the 4-in-1 resection block. **D** Use the AP axis as an additional visual reference to confirm the external rotation resection. **E** Install the 4-in-1 resection block. **F** A well-balanced flexion gap equal to the extension gap was obtained

**Fig. 3:**
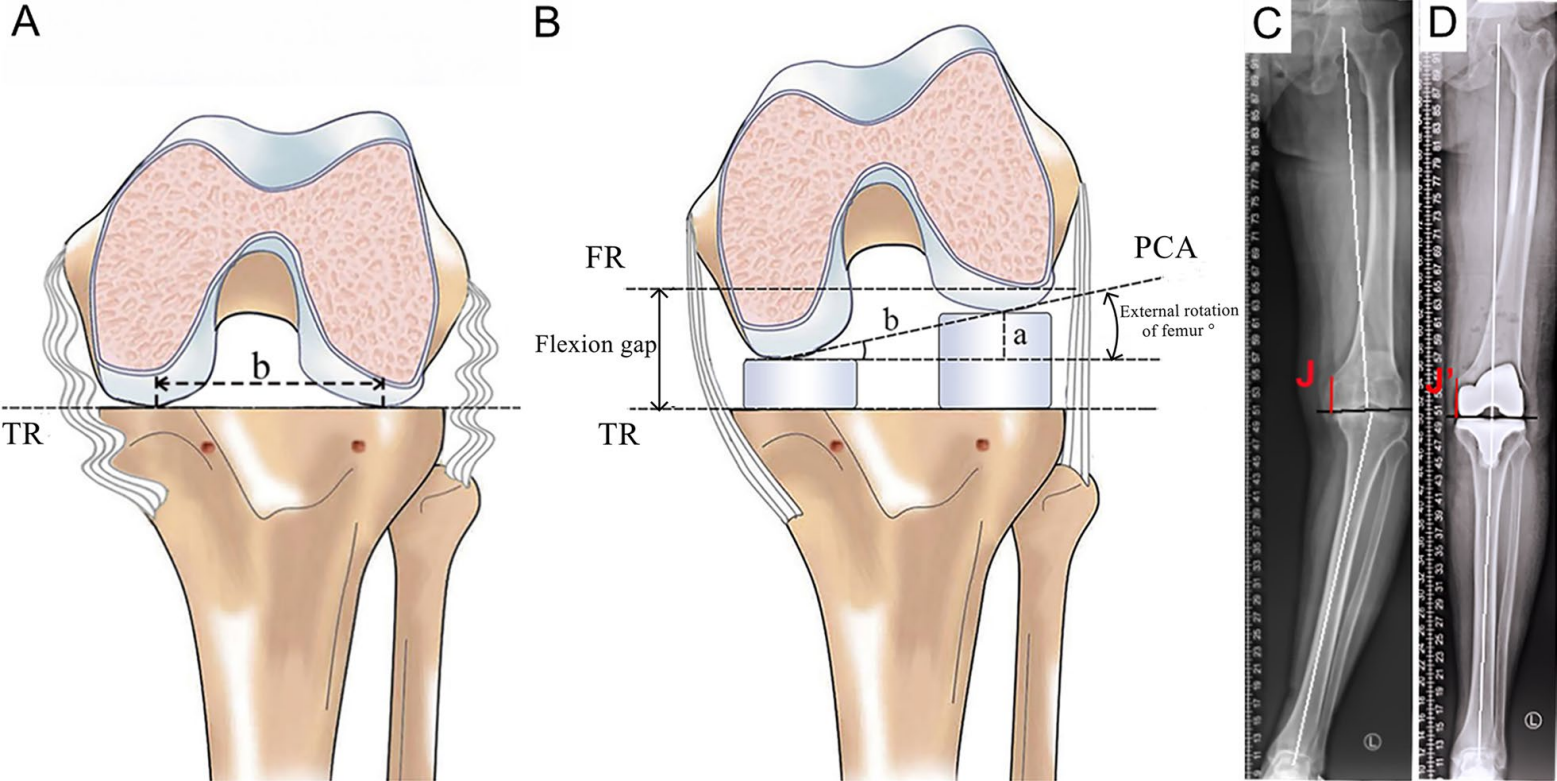
Schematic diagram of measurement results. **A** The knee was flexed 90°, tension was not applied on both sides of the medial and lateral compartment in flexion after the tibial resection was performed. **B** The modified spacers were inserted into the medial and lateral joint space in flexion to balance the tension of the medial and lateral ligaments, and the external rotation angle of femur was also determined. **C** and **D** Measurements of preoperative and postoperative J and the HKA° in full-length anteroposterior X-rays film. TR tibial resection line, FR posterior femoral condyles resection line, **a** The difference between the thickness of the medial and lateral spacers, **b** The distance between the farthest points of the posterior bicondylar, PCA the posterior condyle axis, The external rotation angle of femur = Arcsine a/b. J, J’(red line) is the length from the adductor tubercle to the joint line (black line), The joint line displacement = preoperative J − postoperative J’. The HKA ° (white line), the angle between the center of the femoral head to the center of the knee and the center of the knee to the center of the ankle

After completing the distal femur and proximal tibia resection, sequential medial releases were performed as required to create a rectangular extension gap, which was verified by inserting a 19-mm traditional spacer block (Fig. 2A). Then, the knee was flexed 90°. The measuring units of the modified spacer blocks were sequentially inserted into the medial and lateral joint space in order from small to large. As the medial and lateral joint spaces were stretched, the tension on the medial and lateral ligaments gradually increased. When the tension of the medial and lateral ligaments were balanced (under valgus stress, the medial compartment was stretched within 1 mm, and under varus stress, the lateral compartment was allowed to be stretched by 1–2 mm; Fig. 2B), the thickness of the two spacers determined the external rotation resection angle of the femur (the angle between the PCA and the cut tibial surface, Fig. 3B). Then, a specially designed condylar measuring device was placed on the handle of the two spacers. Two temporary nails were driven into the nail holes of the device (refer to the thickness of the expansion gap that has been obtained, such as mark “19”) to determine the AP position of the 4-in-1 resection block, and the size of the femoral component was obtained by measuring the AP diameter of the condyle (Fig. 2C). We also used the AP axis as an additional visual reference to confirm the component rotation (Fig. 2D). Next, an appropriately sized 4-in-1 resection block was installed in the nail hole (Fig. 2E), and the block was utilized to perform anterior, posterior, and chamfer bone cuts. Finally, a rectangular flexion gap equivalent to the extension gap was obtained (Fig. 2F). No soft tissue was released after this step. The thickness of the medial and lateral posterior condylar bone resection was measured with callipers in both groups.

### MR group

The traditional posterior condylar referencing jig was conventionally set to 3° of external rotation to the PCA to determine the rotation of the femur [23]. In this process, we paid special attention to whether the posterior condyle had hypoplasia or erosion and rechecked that rotation with the AP or TEA. After cutting the anterior, posterior, and chamfered bones with the appropriate 4-in-1 resection block, the ligaments were released as needed to balance the knee.

The following procedures for the two groups were the same, mainly including the processing of the tibial keel, patella and patella trajectory.

### Postoperative management

No patients had drainage tubes placed. Prophylactic antibiotics were intravenously administered within 24 h after surgery. Rivaroxaban (10 mg/day) was orally administered for 21 days to prevent deep venous thrombosis in the lower extremities. Two days after the operation, the patient began to take the initiative to perform quadriceps muscle contractions, CPM training, and ambulation with a walking aid.

### Outcome measures

Intraoperative data collection included the operative time, blood loss, thickness of the cut posterior condyle, and the angle of femoral rotation resection relative to the PCA. (This value is the degree of rotation set on the reference jig in the MR group but in the GB group it is calculated by the trigonometric function formula based on the thickness of the medial and lateral modified spacers; Fig. 3B.)

Patients were followed at 6 weeks, 6 months, 1 year, and then annually. Any complication of the treatment was recorded. Each clinical follow-up examination included the range of motion (ROM), knee stability tests (subjective varus–valgus stress testing can be performed to assess the stability of the knee), and full-length AP and lateral X-rays of the knee for radiological evaluation. The hip–knee–ankle angle (HKA, Fig. 3C), medial distal femoral angle (MDFA, the angle between the distal articular surface and the mechanical axis of the femur), medial proximal tibial angle (MPTA, the angle between the proximal articular surface and the mechanical axis of the tibia) and joint line displacement [24] (Fig. 3D) were measured by one reviewer blinded to the surgical technique using a picture archiving and communication system (PACS) presenting the preoperative and postoperative radiographs. Patients whose mechanical axial alignment was not in the range of 180.0° ± 3.0° were considered as HKA outliers [25]. In addition, the Knee Society Score (KSS) and the Western Ontario and McMaster Universities Osteoarthritis Index (WOMAC) scores of patients preoperatively and postoperatively at 6 weeks, 6 months and 2 years were recorded.

### Statistical analysis

All statistical analyses were performed using IBM SPSS software version 21.0 (Chicago, IL, USA). Categorical variables were compared with the Chi-square or Fisher’s exact test. Student’s t test and Mann–Whitney U test were adopted for continuous variables with normal and nonnormal distributions to analyze the differences between the two groups. Statistical significance was set at *P* < 0.05.

## Results

### Patient outcomes

All patients were followed up. The follow-up time ranged from 36 to 53 months, with an average of 39 months. There were no significant differences in the ROM, KSS scores or WOMAC scores between the groups at 6 weeks, 6 months, or 2 years post-surgery (Table 2).

**Table 2:** Comparison of clinical parameters

Parameters	GB group (*n* = 61)	MR group (*n* = 63)	*P* value
Preoperative HKA (°)	169.62 ± 3.10	170.68 ± 3.40	0.082
Postoperative HKA	178.65 ± 1.30	178.34 ± 1.71	0.275
Outliers (> 3.0°) (%)	5 (8.20)	6 (9.52)	0.795
Preoperative MDFA (°)	90.49 ± 2.41	91.08 ± 2.61	0.195
Postoperative MDFA (°)	90.14 ± 1.18	90.31 ± 1.51	0.495
Preoperative MPTA (°)	83.74 ± 1.96	84.20 ± 2.19	0.251
Postoperative MPTA (°)	89.65 ± 1.09	89.40 ± 1.27	0.253
The joint line displacement (mm)	1.38 ± 0.90	1.20 ± 0.87	0.270
Number of complications (%)	2 (3.28)	3 (4.76)	0.515
Preoperative ROM (°)	96.07 ± 13.23	94.68 ± 13.29	0.563
Postoperative ROM at 6 weeks	98.85 ± 7.15	99.60 ± 7.03	0.557
Postoperative ROM at 6 months	109.67 ± 8.44	108.17 ± 8.81	0.347
Postoperative ROM at 2 years	114.02 ± 12.10	111.67 ± 11.91	0.278
Preoperative KSS	44.54 ± 13.48	45.71 ± 12.66	0.618
Postoperative KSS at 6 weeks	75.16 ± 7.67	75.21 ± 8.13	0.976
Postoperative KSS at 6 months	88.33 ± 4.01	87.59 ± 3.61	0.281
Postoperative KSS at 2 years	94.92 ± 4.50	94.21 ± 4.49	0.379
Preoperative WOMAC	60.30 ± 10.11	62.76 ± 9.42	0.162
Postoperative WOMAC at 6 weeks	36.80 ± 6.96	37.84 ± 7.03	0.411
Postoperative WOMAC at 6 months	26.03 ± 3.74	26.94 ± 4.28	0.213
Postoperative WOMAC at 2 years	9.64 ± 4.10	10.0 ± 3.94	0.618

HKA, Hip–knee–ankle angle; MDFA, the angle between the distal articular surface and the mechanical axis of the femur; MPTA, the angle between the proximal articular surface and the mechanical axis of the tibia; ROM, range of motion; KSS, knee society score; WOMAC, Western Ontario and McMaster Universities Osteoarthritis Index

### Complications

No patients in either group had undergone revision surgery by the end of the last follow-up. After the surgery, complications of knee anterior pain (2 knees) occurred in the GB group (2/61), and slight postoperative knee laxity (1 knee), knee anterior pain (1 knee), and periprosthetic joint infection (1 knee) occurred in the MR group (3/63). There was no difference in the number of complications between the two groups (Table 2). The postoperative knee laxity patient in the MR group showed slight valgus laxity in flexion, but he did not complain of knee instability, and his clinical score was similar to that of the other patients. One patient developed an acute infection 28 days after implantation and was treated with debridement and implant retention (DAIR), and he had recovered at the 6-month follow-up. Three patients with anterior knee pain caused by patellar arthritis were relieved after taking Celebrex capsules for 3 months.

### Radiographic analysis

The mean value of the postoperative mechanical axis (HKA°) was 178.65° ± 1.30° in the GB group and 178.35° ± 1.71° in the MR group (*P* = 0.275). There was no significant difference in the number of HKA outliers between the two groups (*P* = 0.795). The postoperative MDFA (*P* = 0.495) and MPTA (*P* = 0.253) were similar between the groups. There was also no significant difference in the joint line displacement between the groups (*P* = 0.270, Table 2).

### Surgical outcomes

There were no significant differences between the GB and MR groups in terms of the operative time (*P* = 0.075) or intraoperative blood loss (*P* = 0.251, Table 3). The angle of femoral rotation resection relative to PCA in the GB group was statistically greater than that in the MR group (4.06 ± 1.10° vs. 3.19 ± 0.59°, *P* < 0.001). The thickness of the cut posterior medial condyle was larger than that in the MR group (9.72 ± 0.84 mm vs. 9.25 ± 0.62 mm, *P* = 0.001). In contrast, the thickness of the cut posterior lateral condyle was similar in the two groups, measuring 6.91 ± 0.71 mm in the GB group and 7.08 ± 0.53 mm in the MR group (*P* = 0.137, Table 3).

**Table 3:** Intraoperative outcome

	GB group (*n* = 61)	MR group (*n* = 63)	*P* value
Operative time (min)	76.25 ± 12.43	80.25 ± 12.44	0.075
Blood loss (ml)	55.33 ± 11.06	57.78 ± 12.50	0.251
External rotation of femur (°) relative to PCA	4.06 ± 1.10	3.19 ± 0.59	< 0.001
Posterior medial condyle cut thickness (mm)	9.72 ± 0.84	9.25 ± 0.62	0.001
Posterior lateral condyle cut thickness (mm)	6.91 ± 0.71	7.08 ± 0.53	0.135

## Discussion

In this study, we evaluated the clinical effects of the gap balancing technique based on a modified spacer block and measured the resection technique through intraoperative indicators, postoperative X-ray findings, and clinical scores.

One of the important findings was that the GB technique did not result in better functional outcomes or clinical scores than the MR technique at the 3-year follow-up. The ROM, KSS scores and WOMAC scores between the two groups were very similar. Our results are consistent with those of several other studies. Moorthy et al. [26] came to a similar conclusion after conducting a randomized controlled trial. They found no significant differences in the functional scores or the proportion of patients between the gap balancing and measured resection groups who were satisfied at 6-month or 2-year post-surgery. Similar results were also reported by Deng et al. [27]. They conducted a retrospective study and concluded that gap balancing performed with a new balancing device and PSI could produce accurate femoral component alignment as well as outcomes similar to those of the measured resection technique at 3 years. Previous researchers [11] confirmed that there were indeed technical differences between GB and MR technology, but it was difficult to detect any consistent superiority of either technology by using functional outcome scores.

Another important finding was that the angle of femoral rotation resection relative to PCA of the GB group (4.06 ± 1.10) was greater than that of the MR group (3.19 ± 0.59, *P* < 0.05). While studies have reported that a relative external rotation of 3 or 4 degrees relative to the PCA will orient the AP femoral bone resections perpendicular to the resected tibial surface [8], other data have shown a wide anatomic variation in the relationship of the posterior condylar axis to the TEA [28]. Moon et al. and others have noticed that gap balancing technology leads to more external rotation than measured resection technology [29, 30]. In addition, Yau et al. [31] reported larger medially inclined (5° ± 3°) and posterior condyle angles (5° ± 2°) of the knee for Chinese patients than for Caucasians. From this, we infer that this is also one of the reasons for the increase in the femoral external rotation in the GB group. Another important reason for abnormal femoral rotation with GB technology is that the tibial cut is not perpendicular to the tibial mechanical axis. Therefore, we were careful with our resection technique when cutting the proximal tibia. The average value of postoperative MPTA in both groups was close to 90°. In the GB group, there were two patients with anterior knee pain, but it was found that the source of pain was not a poor patellar trajectory but patellar arthritis.

We also found that the bone resection from the posterior medial condyle in the GB group was larger than that in the MR group (9.72 ± 0.84 mm vs. 9.25 ± 0.62 mm, *P* = 0.001). Several studies have reached similar conclusions on this point. In a comparative study of GB and MR techniques in patients undergoing simultaneous bilateral TKA, Tapasvi et al. [11] found that the GB technique requires a larger bone resection from the posterior medial femur to achieve a rectangular flexion gap. The resection of the posterior condyle with the GB technique is greater than that with the MR technique, which has been confirmed by Cidambi et al. [6]. It is worth mentioning that an increase or decrease in posterior condylar bone resection leads to poor recovery of the posterior condylar offset (PCO), which is one of the reasons for post-surgery flexion instability after TKA [32]. However, we found no cases of postoperative flexion instability in the GB group.

Longo et al. proposed that if the joint line position changes within the maximum range of 5 mm, the knee stability will not change significantly [33]. In this study, the joint line displacement of the two groups was similar, and no cases where the position of the joint line changed more than 5 mm were found.

In our research, gap balancing technology based on spacer blocks was chosen instead of tension devices because we believe that the tools of our modified spacer block have some additional advantages. First, it has a low cost, a simple structure and a low probability of intraoperative failure. Second, it simulates the restoration of a normal knee joint by temporarily replacing the cut bone to obtain a more natural ligament balance. Third, the implementation procedure of using spacer block tools in GB technology is not as complicated as other tension devices or even computer-assisted navigation. The surgeon does not have to actively consider how much tension should be applied but passively feels the pressure released by the soft tissue to adjust the balance of the flexion gap, so we believe that the application of the spacer block tool is simpler and more flexible. In addition, there is no need to release soft tissue after the completion of post-condylar resection with GB technology. Therefore, we infer that combining these two favorable factors can reduce intraoperative trauma and the operation time, which is beneficial to patients in early recovery. In this study, although the average operation time and blood loss of the GB group were lower than those of the MR group, there were no significant differences between the two groups, and more cases need to be observed.

Our study had several limitations. First, there is a lack of accurate mechanical quantitative indicators; therefore, the size of the modified spacer relies entirely on the surgeon's experience. Second, this was a retrospective study. The postoperative TKA position was not evaluated with computed tomography (CT) scans, and we could not compare the femoral component rotation angle with bony markers. Third, it is not clear whether the new spacer gap balancing tool can be used in cases of severe varus and valgus or even severe extra-articular deformities, so additional research is necessary in these cases. Finally, although patients in this study had a minimum follow-up of 3 years, a better survival rate of the prosthesis and a more comprehensive understanding of the effect of the two surgical techniques can be observed over a longer term.

## Conclusion

This study shows that the functional outcomes of the gap balancing technique based on the modified spacer are similar to those of measured resection at 3 years. Compared with the MR technique, the GB technique requires a greater external rotation resection angle of the femur and more posterior femoral condyle resections in the application of TKA with knee varus. This set of innovative and convenient spacer block tools can be taken into consideration by surgeons who prefer gap balancing techniques.

## Data Availability

The datasets generated and analyzed during the current study are available from the corresponding author on reasonable request.

## Abbreviations

TKA: Total knee arthroplasty
GB: Gap balancing
MR: Measured resection
TEA: Transepicondylar axis
AP: Anteroposterior
PCA: Posterior condyle axis
ROM: Range of motion
HKA: Hip–knee–ankle angle
MDFA: Medial distal femoral angle
MPTA: Medial proximal tibial angle
CAS: Computer assisted navigation
KSS: Knee society score
WOMAC: Western Ontario and McMaster Universities Osteoarthritis Index

## References

[1] Siddiqi A, Smith T, McPhilemy JJ, Ranawat AS, Sculco PK, Chen AF (2020). Soft-tissue balancing technology for total knee arthroplasty. JBJS Rev.

[2] Bourne RB, Chesworth BM, Davis AM, Mahomed NN, Charron KD (2010). Patient satisfaction after total knee arthroplasty: who is satisfied and who is not?. Clin Orthop Relat Res.

[3] Le DH, Goodman SB, Maloney WJ, Huddleston JI (2014). Current modes of failure in TKA: infection, instability, and stiffness predominate. Clin Orthop Relat Res.

[4] Lombardi AV, Berend KR, Adams JB (2014). Why knee replacements fail in 2013: patient, surgeon, or implant?. Bone Joint J.

[5] Sheth NP, Husain A, Nelson CL (2017). Surgical techniques for total knee arthroplasty: measured resection, gap balancing, and hybrid. J Am Acad Orthop Surg.

[6] Cidambi KR, Robertson N, Borges C, Nassif NA, Barnett SL (2018). Intraoperative comparison of measured resection and gap balancing using a force sensor: a prospective. Randomized Controlled Trial J Arthroplasty.

[7] Mercuri JJ, Pepper AM, Werner JA, Vigdorchik JM (2019). Gap balancing, measured resection, and kinematic alignment: how, when, and why?. JBJS Rev.

[8] Daines BK, Dennis DA (2014). Gap balancing vs. measured resection technique in total knee arthroplasty. Clin Orthop Surg..

[9] Springer BD, Parratte S, Abdel MP (2014). Measured resection versus gap balancing for total knee arthroplasty. Clin Orthop Relat Res.

[10] Castelli CC, Falvo DA, Iapicca ML, Gotti V (2016). Rotational alignment of the femoral component in total knee arthroplasty. Ann Transl Med.

[11] Tapasvi SR, Shekhar A, Patil SS, Dipane MV, Chowdhry M, McPherson EJ (2020). Comparison of gap balancing vs measured resection technique in patients undergoing simultaneous bilateral total knee arthroplasty: one technique per knee. J Arthroplasty.

[12] Franceschini V, Nodzo SR, Gonzalez Della Valle A (2016). Femoral component rotation in total knee arthroplasty: a comparison between transepicondylar axis and posterior condylar line referencing. J Arthroplasty..

[13] Yau WP, Chiu KY, Tang WM (2007). How precise is the determination of rotational alignment of the femoral prosthesis in total knee arthroplasty: an in vivo study. J Arthroplasty.

[14] Dennis DA, Komistek RD, Kim RH, Sharma A (2010). Gap balancing versus measured resection technique for total knee arthroplasty. Clin Orthop Relat Res.

[15] Grifka J, Baier C, Maderbacher G (2020). Improved femoral component rotation in total knee arthroplasty: an anatomical study with optimized gap balancing. Arch Orthop Trauma Surg.

[16] Babazadeh S, Dowsey MM, Stoney JD, Choong PF (2014). Gap balancing sacrifices joint-line maintenance to improve gap symmetry: a randomized controlled trial comparing gap balancing and measured resection. J Arthroplasty.

[17] Lavoie F (2017). Spacer-based gap balancing in total knee arthroplasty: clinical success with a reproducible technique. J Knee Surg.

[18] Nagai K, Muratsu H, Matsumoto T, Miya H, Kuroda R, Kurosaka M (2014). Soft tissue balance changes depending on joint distraction force in total knee arthroplasty. J Arthroplasty.

[19] Cheng T, Zhang G, Zhang X (2011). Imageless navigation system does not improve component rotational alignment in total knee arthroplasty. J Surg Res.

[20] Parratte S, Blanc G, Boussemart T, Ollivier M, Le Corroller T, Argenson JN (2013). Rotation in total knee arthroplasty: no difference between patient-specific and conventional instrumentation. Knee Surg Sports Traumatol Arthrosc.

[21] Bercovy M, Kerboull L, Müller JH, Saffarini M, Sailhan F. Satisfactory mid-to long-term outcomes of TKA aligned using conventional instrumentation for flexion gap balancing with minimal soft tissue release. Knee Surg Sports Traumatol Arthrosc. 2020; 10.1007/s00167-020-06360-3. 10.1007/s00167-020-06360-3 33175282

[22] Gungor HR, Ok N. Use of a spacer block tool for assessment of joint line position during revision knee arthroplasty. J Knee Surg. 2021; 10.1055/s-0040-1722628. 10.1055/s-0040-1722628 33472259

[23] Bottros J, Gad B, Krebs V, Barsoum WK (2006). Gap balancing in total knee arthroplasty. J Arthroplasty.

[24] Yeh KT, Chen IH, Wang CC, Wu WT, Liu KL, Peng CH (2019). The adductor tubercle can be a radiographic landmark for joint line position determination: an anatomic-radiographic correlation study. J Orthop Surg Res.

[25] Longstaff LM, Sloan K, Stamp N, Scaddan M, Beaver R (2009). Good alignment after total knee arthroplasty leads to faster rehabilitation and better function. J Arthroplasty.

[26] Moorthy V, Lai MC, Liow MHL (2020). Similar postoperative outcomes after total knee arthroplasty with measured resection and gap balancing techniques using a contemporary knee system: a randomized controlled trial. Knee Surg Sports Traumatol Arthrosc..

[27] Deng T, Liu T, Lei Q, Cai L, Chen S (2021). Patient-specific instrumentation combined with a new tool for gap balancing is useful in total knee replacement: a 3-year follow-up of a retrospective study. J Orthop Surg Res..

[28] Poilvache PL, Insall JN, Scuderi GR, Font-Rodriguez DE (1996). Rotational landmarks and sizing of the distal femur in total knee arthroplasty. Clin Orthop Relat Res.

[29] Moon YW, Kim HJ, Ahn HS, Park CD, Lee DH (2016). Comparison of soft tissue balancing, femoral component rotation, and joint line change between the gap balancing and measured resection techniques in primary total knee arthroplasty: a meta-analysis. Medicine (Baltimore).

[30] Luyckx T, Peeters T, Vandenneucker H, Victor J, Bellemans J (2012). Is adapted measured resection superior to gap-balancing in determining femoral component rotation in total knee replacement?. J Bone Joint Surg Br.

[31] Yau WP, Chiu KY, Fok AW, Yan CH, Ng FY (2013). Distal femur rotation relates to joint obliquity in ACL-deficient Chinese. Clin Orthop Relat Res.

[32] Clarke HD, Scuderi GR (2003). Flexion instability in primary total knee replacement. J Knee Surg.

[33] Longo UG, Candela V, Pirato F, Hirschmann MT, Becker R, Denaro V (2021). Midflexion instability in total knee arthroplasty: a systematic review. Knee Surg Sports Traumatol Arthrosc.

